# Immune checkpoint inhibitors in pancreatic adenocarcinoma: a systematic review and meta analysis of clinical outcomes

**DOI:** 10.3389/fonc.2025.1569884

**Published:** 2025-08-08

**Authors:** Aisha Al-Khinji, Noora Al-Korbi, Sheikha Al-Kuwari, Abdullatif Al-Hor, Dhafer Malouche

**Affiliations:** ^1^ College of Medicine, Qatar University, Doha, Qatar; ^2^ Clinical Translational Science Research Group, Qatar University (QU) Health, Qatar University, Doha, Qatar; ^3^ Department of Mathematics and Statistics, College of Arts and Sciences, Qatar University, Doha, Qatar

**Keywords:** pancreatic ductal adenocarcinoma (PDAC), immune checkpoint inhibitors (ICIs), tumor mutational burden, combination therapy, survival outcomes

## Abstract

**Background:**

Pancreatic ductal adenocarcinoma (PDAC) remains one of the most aggressive malignancies, with poor outcomes despite therapeutic advancements. Immune checkpoint inhibitors (ICIs) have transformed cancer care, but their efficacy in PDAC is limited due to the tumor’s immunosuppressive microenvironment.

**Methods:**

We systematically reviewed and meta-analyzed clinical outcomes of ICI therapy in PDAC using studies from PubMed, CINAHL, Cochrane Library, and Google Scholar, published up to February 28, 2024. Eligible studies reported objective response rate (ORR), progression-free survival (PFS), or overall survival (OS). Risk of bias was assessed using RoB 2.0 and ROBINS-I. Random-effects models estimated pooled effect sizes.

**Results:**

Fifty-four studies (*n* = 2,364) were included. ORR ranged from 0% to 67%. ICI-based combinations showed a modest ORR benefit (OR = 1.10; 95% CI: 1.02–1.18) and improved OS when combined with chemotherapy (HR = 0.82; 95% CI: 0.78–0.87). However, ICIs plus radiotherapy were associated with increased mortality (HR = 1.18; 95% CI: 1.04–1.34). PFS improved in select subgroups, particularly in patients with high tumor mutational burden or mismatch repair deficiency.

**Conclusion:**

ICIs combined with chemotherapy may modestly improve survival in PDAC. Outcomes remain heterogeneous and limited, underscoring the need for better biomarker-driven patient selection and more effective combination strategies.

## 1 Introduction

Pancreatic adenocarcinoma (PDAC) is among the most lethal and challenging cancers to treat, with limited improvements in survival despite decades of research and clinical advancements [see (1–3)]. Epidemiological studies, such as that by Neoptolemos et al. (4), highlight the poor prognosis associated with PDAC, with five-year survival rates remaining below 2%. This underscores the critical need for effective therapeutic strategies to address this devastating disease.

The molecular and genetic underpinnings of PDAC have been extensively studied, revealing key drivers such as mutations in KRAS, TP53, and CDKN2A [see (5, 6)]. These genetic alterations contribute to the aggressive biology of PDAC, including its dense stromal microenvironment and immunosuppressive characteristics (7, 8). The tumor microenvironment (TME), characterized by high collagen density, fibrotic stroma, and abundant immunosuppressive cells (e.g., regulatory T cells, myeloid-derived suppressor cells), further complicates treatment by promoting therapy resistance, excluding effector immune cells, and limiting drug delivery (7, 9). This “cold” immune milieu with low antigen presentation and limited T-cell infiltration is a key reason for the poor response to immune checkpoint blockade in PDAC. These factors collectively hinder the efficacy of traditional therapies, including surgery, chemotherapy, and radiation, and present a substantial challenge for immunotherapy strategies.

The emergence of immunotherapy, particularly immune checkpoint inhibitors (ICIs), has revolutionized the treatment of various cancers by harnessing the immune system to target and destroy tumor cells [see (10, 11)]. However, in pancreatic cancer, single-agent immunotherapies have generally yielded limited success. Royal et al. (12) showed that the immunosuppressive tumor microenvironment and low mutational burden are major barriers to the efficacy of ICIs in PDAC. Similarly, Quintanilha et al. (13) found that tumor mutational burden and genomic alterations play a critical role in predicting the effectiveness of ICIs. Despite these challenges, there is growing interest in combination therapies that integrate ICIs with other modalities, such as chemotherapy, radiation, targeted therapies, and immunomodulators [see (14, 15)]. These approaches aim to prime the immune system, disrupt tumor defense mechanisms, and overcome resistance to immunotherapy.

Early-phase clinical trials have shown some encouraging results, suggesting that combination therapies may enhance the efficacy of ICIs in PDAC. For example, Anderson et al. (16) demonstrated that combining ICIs with chemotherapy could improve clinical outcomes in certain patient subgroups. Similarly, O’Reilly et al. (17) reported that perioperative chemotherapy significantly enhances survival outcomes for resectable PDAC. However, conflicting outcomes persist, often influenced by variations in study designs, patient populations, and treatment regimens [see (18)]. This underscores the need for a systematic appraisal of the evidence to evaluate the effectiveness of ICIs in PDAC, clarify their role in clinical practice, and guide future research directions.

This study systematically investigates the impact of immune checkpoint inhibitors on key clinical outcomes—specifically progression-free survival (PFS), overall survival (OS), and objective response rate (ORR)—in patients with pancreatic adenocarcinoma. By synthesizing the available evidence, this review aims to provide a comprehensive understanding of the current state of immunotherapy in PDAC, identify gaps in the literature, and offer insights into optimizing treatment strategies for this challenging disease.

## 2 Methods

### 2.1 Literature search

A systematic search of PubMed, Embase, Scopus, Web of Science, and ClinicalTrials.gov was conducted from inception to February 28, 2024. The search combined MeSH and free-text terms related to “pancreatic cancer” and “immune checkpoint inhibitors” (ICIs). Full search strings used for each database are provided in **Supplementary Table 1**.

### 2.2 Study design

This systematic review and meta-analysis adhered to the Preferred Reporting Items for Systematic Reviews and Meta-Analyses (PRISMA) guidelines (19). The study aimed to evaluate the clinical outcomes of immune checkpoint inhibitors (ICIs) in pancreatic adenocarcinoma (PDAC), focusing on progression-free survival (PFS), overall survival (OS), and objective response rate (ORR).

### 2.3 Search strategy

A comprehensive literature search was conducted across multiple databases, including PubMed, CINAHL Open Research, Cochrane Library, and Google Scholar, up to [insert date of search]. The search strategy utilized a combination of keywords and Medical Subject Headings (MeSH) terms related to immune checkpoint inhibitors, pancreatic adenocarcinoma, and clinical outcomes (see **Supplementary Table 1** for the full search strings). The Rayyan tool (20) was employed to manage and screen the search results.

### 2.4 Inclusion and exclusion criteria

Studies were selected based on predefined eligibility criteria, modified from the PICOS framework (21):

- Population: Patients diagnosed with pancreatic adenocarcinoma (PDAC).
- Intervention: Immune checkpoint inhibitors (ICIs), either as monotherapy or in combination with other treatments (e.g., chemotherapy, radiotherapy).
- Comparison: Standard treatments (e.g., chemotherapy alone) or placebo.
- Outcomes: Progression-free survival (PFS), overall survival (OS), and objective response rate (ORR).
- Study Design: Randomized controlled trials (RCTs), phase Ib/II/III trials, retrospective studies, and observational studies.

Studies were excluded if they were reviews, meta-analyses, conference abstracts, letters, editorials, or opinion pieces. Additionally, studies involving animal models or non-human subjects were excluded.

### 2.5 Study selection and data extraction

**Figure 1:**
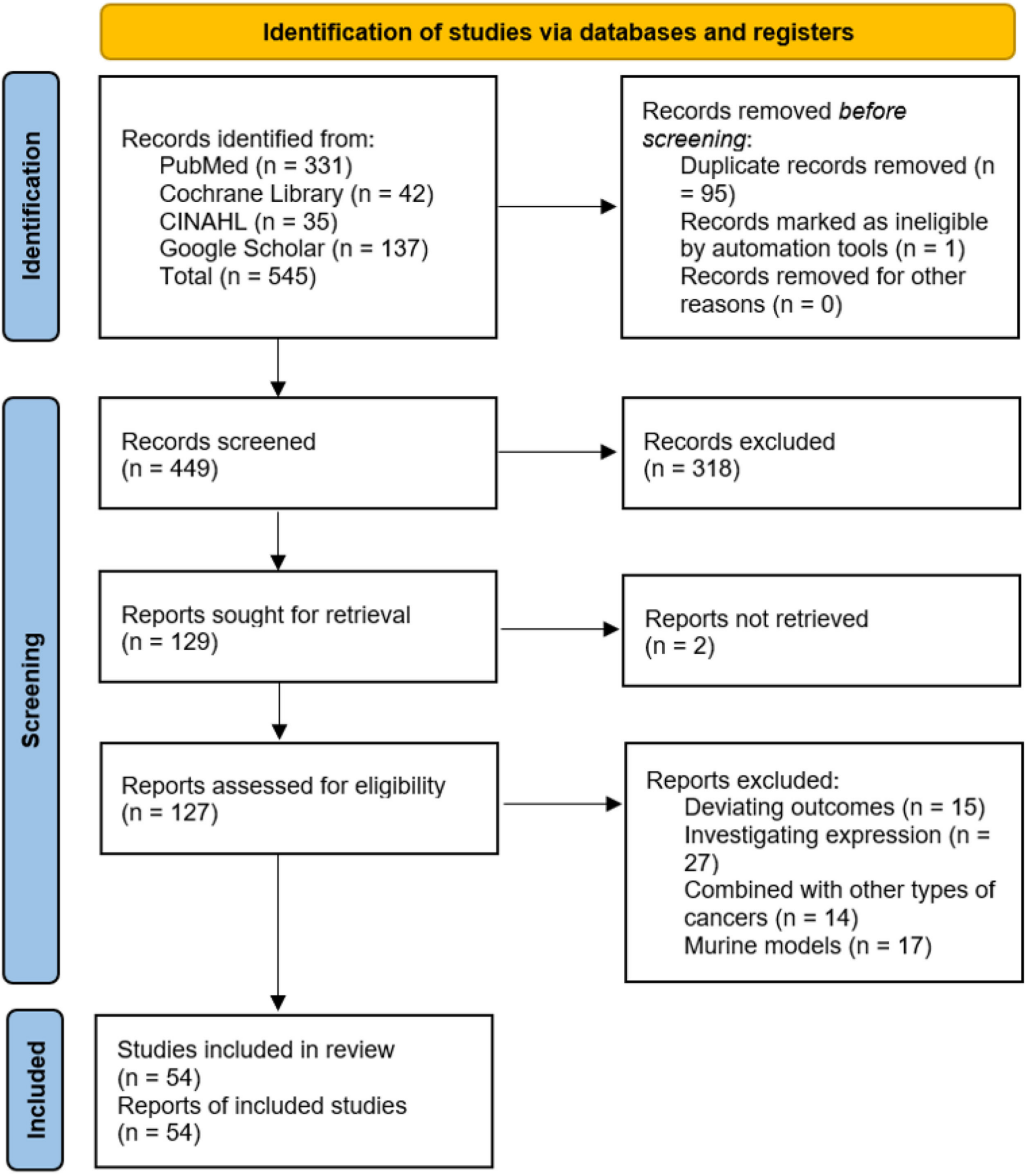
PRISMA flow diagram illustrating the process of study selection for inclusion in the systematic review and meta-analysis.

The study selection process followed the PRISMA flow diagram (see **Figure 1**). Two independent reviewers screened titles and abstracts for eligibility, followed by full-text review of potentially relevant studies. Discrepancies were resolved through discussion or consultation with a third reviewer. Data extraction was performed using a standardized form, capturing study characteristics (e.g., author, year, study design, sample size), intervention details (e.g., type of ICI, combination therapies), and clinical outcomes (e.g., PFS, OS, ORR).

### 2.6 Quality assessment and risk of bias

Risk of bias was assessed using the Cochrane Risk of Bias 2.0 (RoB 2.0) tool for randomized controlled trials (22) and the Risk of Bias in Non-Randomized Studies of Interventions (ROBINS-I) tool for non-randomized studies (23). These tools evaluate key domains of bias, including randomization, deviations from intended interventions, missing data, and outcome measurement. The overall quality of evidence was assessed using the Grading of Recommendations, Assessment, Development, and Evaluations (GRADE) framework (24). Assessment results were visualized using traffic-light plots and considered in the interpretation of pooled results.

### 2.7 Data analysis

Meta-analyses were conducted using RStudio (version 4.4.2) with the meta package Schwarzer (25). Pooled effect sizes for PFS, OS, and ORR were calculated using random-effects models to account for heterogeneity across studies. Heterogeneity was quantified using the Higgins *I* ^2^ statistic, with values of 25%, 50%, and 75% indicating low, moderate, and high heterogeneity, respectively. Forest plots were generated to visualize the pooled effect sizes and their 95% confidence intervals. Sensitivity analyses were performed using the leave-one-out method to assess the robustness of the results. Publication bias was evaluated using funnel plots and Egger’s test (26). A *p*-value of less than 0.05 was considered statistically significant.

### 2.8 Ethical considerations

This study utilized publicly available data from published studies and did not involve direct human or animal subjects. Therefore, ethical approval was not required.

## 3 Results

### 3.1 Study selection process and characteristics

The literature search identified a total of 545 records from PubMed (n = 331), Cochrane Library (n = 42), CINAHL (n = 35), and Google Scholar (n = 137). After removing 95 duplicate records and 1 record marked as ineligible by automation tools, 449 records were screened. Of these, 318 records were excluded based on title and abstract review, leaving 129 reports sought for retrieval. Two reports were not retrieved, and 127 reports were assessed for eligibility. After excluding studies with deviating outcomes (n = 15), those investigating expression (n = 27), studies combining PDAC with other types of cancers (n = 14), and studies involving murine models (n = 17), a total of 54 studies were included in the review. The study selection process is summarized in **Figure 1**.

The included studies comprised 3 single-center open-label trials, 31 phase II/1b trials, and 14 multi-center randomized studies, with a total participant population of 2,364. The sample sizes of the included studies ranged from 3 to 312 participants, reflecting the heterogeneity in trial phases and study designs. The studies compared various immune checkpoint inhibitor (ICI) dosing regimens with standard chemotherapy (e.g., Paclitaxel, Gemcitabine), other ICIs (e.g., Nivolumab/Ipilimumab), and radiotherapy or other modalities such as vaccines. The data estimation point was 12 months after the targeted drug therapy, with varying follow-up periods. Detailed characteristics of the included studies are presented in **Table 1**.

**Table 1:** Detailed search strategy used for the systematic review across PubMed, CINAHL, Cochrane Library, and Google Scholar.

Study	Study design	Sample size	Intervention	Key findings
Ahnert et al. (27)	Phase II	35	Avelumab + Binimetinib	No objective responses observed.
Bassani-Sternberg et al. (28)	Phase Ib	3	Personalized vaccine + Nivolumab	Safe and immunogenic.
Beatty et al. (29)	Open-label	22	CP-870,893 + Gemcitabine	ORR of 19%.
Bockorny et al. (30)	Phase II	43	Motixafortide + Pembrolizumab + Chemo	ORR of 13.2%.
Byrne et al. (31)	Phase I	16	Selicrelumab + Chemo	1-year OS rate of 100%.

### 3.2 Risk of bias assessment

**Figure 2:**
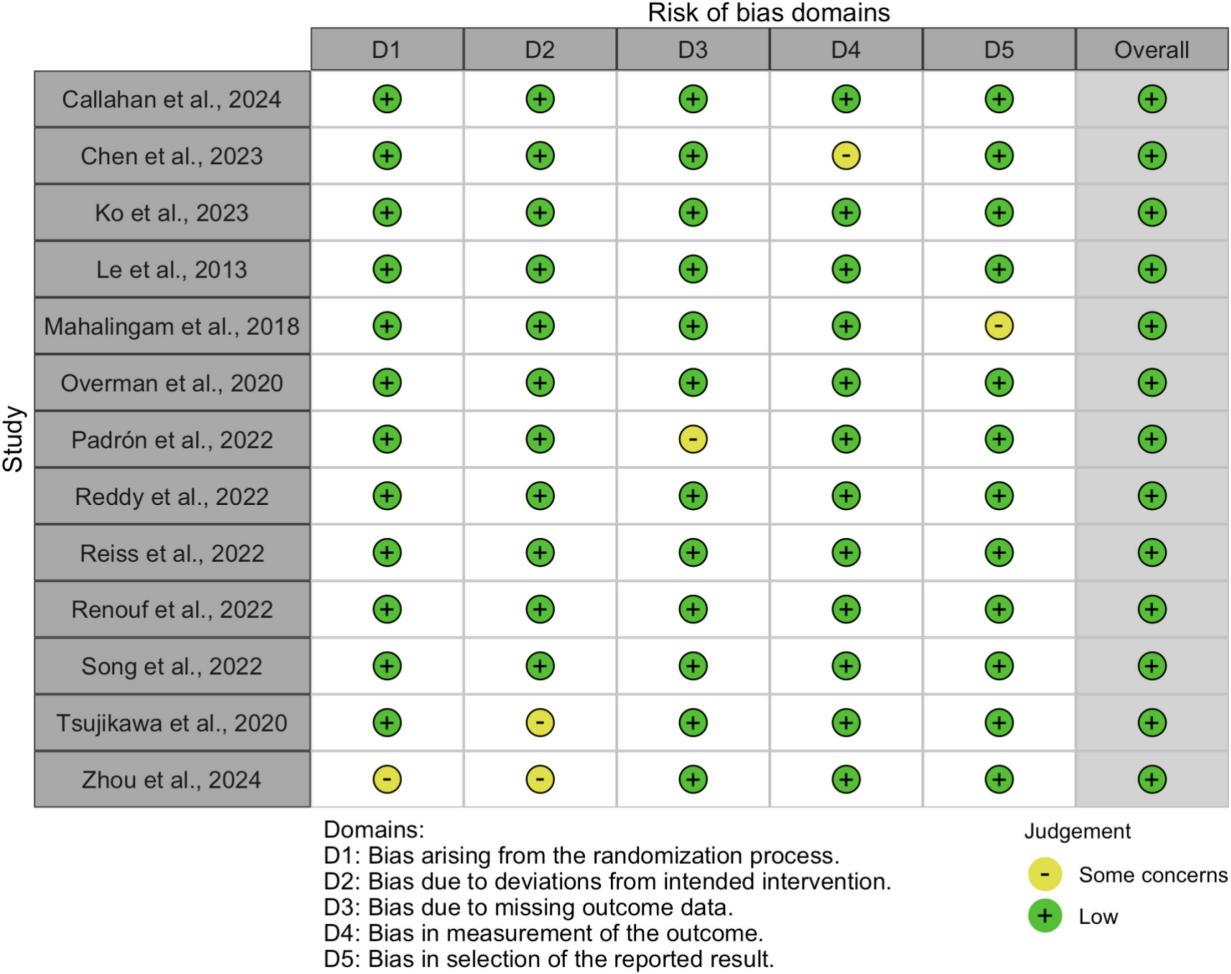
Summary of risk of bias across domains for randomized controlled trials, assessed using the Cochrane RoB 2.0 tool.

**Figure 3:**
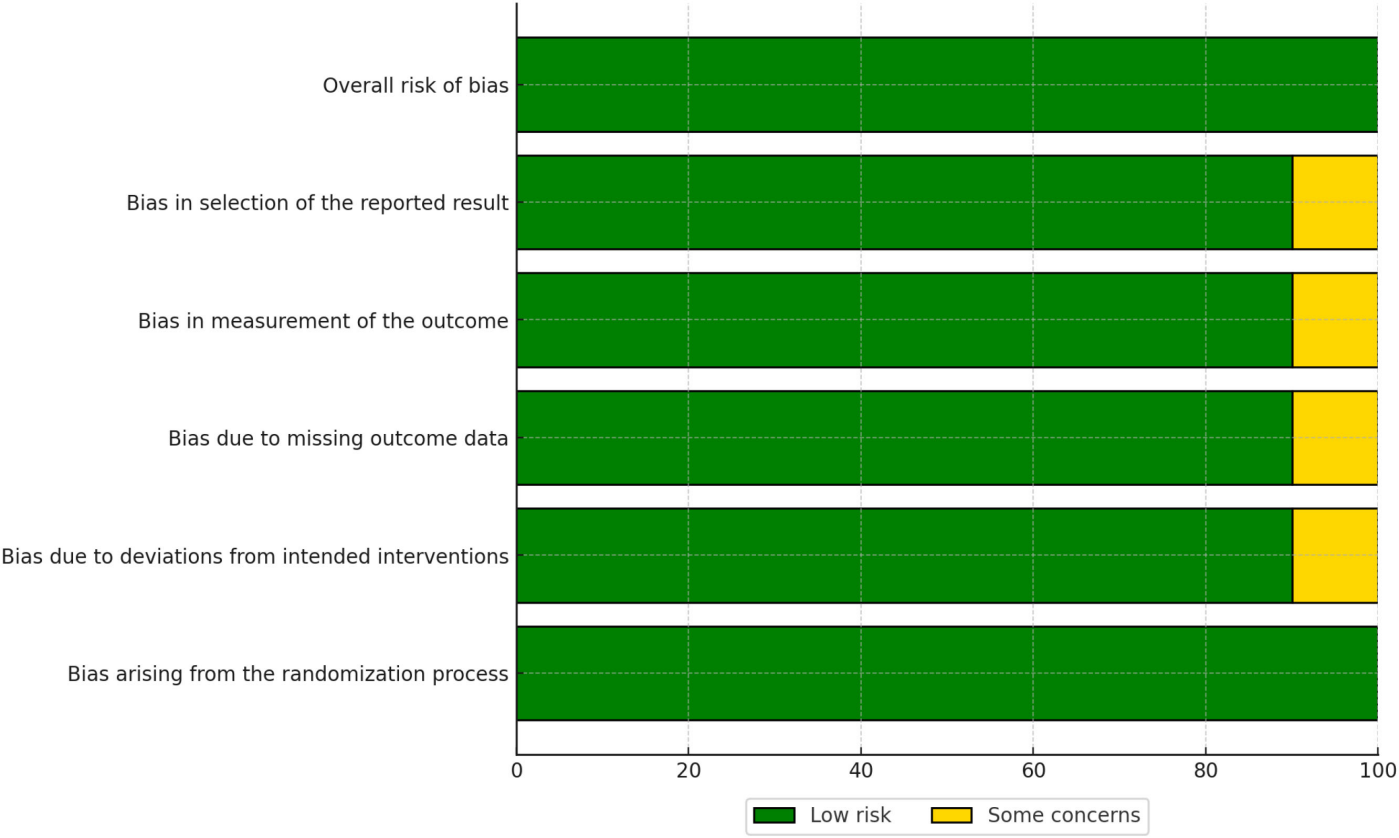
Traffic light plot showing domain-level risk of bias judgments for each included randomized controlled trial.

The risk of bias in the included studies was assessed using the Cochrane Risk of Bias 2.0 (RoB 2.0) tool for randomized controlled trials (RCTs) and the Risk of Bias in Non-Randomized Studies of Interventions (ROBINS-I) tool for non-randomized studies. The results of the risk of bias assessment are summarized in **Figures 2**, **3**.

#### 3.2.1 Randomized controlled trials

For RCTs, the RoB 2.0 tool evaluated five domains of bias:

- Bias arising from the randomization process (D1),
- Bias due to deviations from intended interventions (D2),
- Bias due to missing outcome data (D3),
- Bias in measurement of the outcome (D4), and
- Bias in selection of the reported result (D5).

The overall risk of bias for each RCT is visualized in **Figure 3**. Most RCTs were judged to have a low risk of bias across all domains, although some studies raised concerns in specific areas, such as deviations from intended interventions (D2) and missing outcome data (D3).

#### 3.2.2 Non-randomized studies

For non-randomized studies, the ROBINS-I tool assessed seven domains of bias:

- Bias due to confounding (D1),
- Bias due to selection of participants (D2),
- Bias in classification of interventions (D3),
- Bias due to deviations from intended interventions (D4),
- Bias due to missing data (D5),
- Bias in measurement of outcomes (D6), and
- Bias in selection of the reported result (D7).

**Figure 4:**
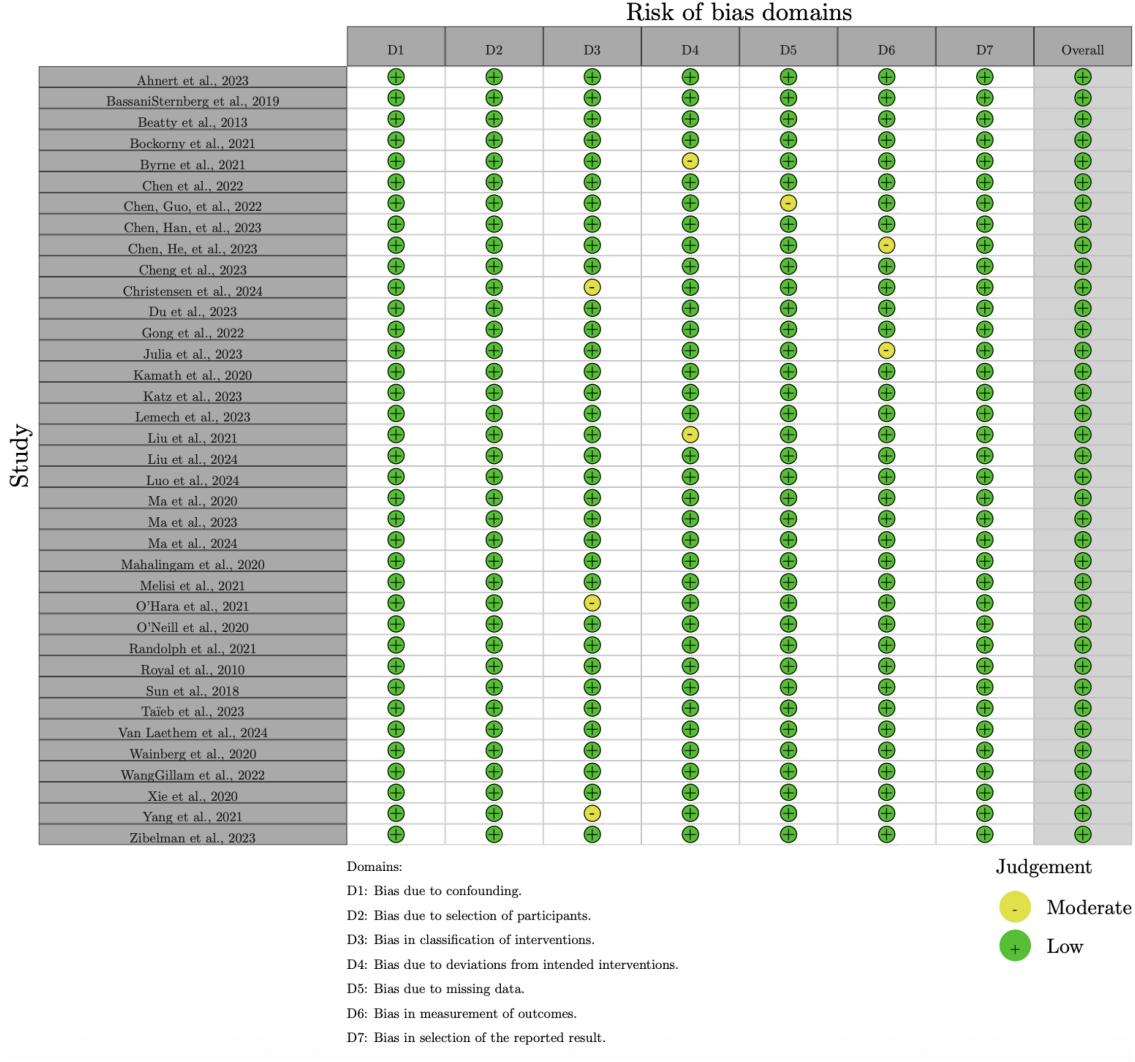
Summary of risk of bias across domains for non-randomized studies, assessed using the ROBINSI tool.

The overall risk of bias for non-randomized studies is presented in **Figure 4**. While many studies were judged to have a moderate risk of bias, some exhibited significant concerns, particularly in the domains of confounding (D1) and selection of participants (D2).

**Figure 5:**
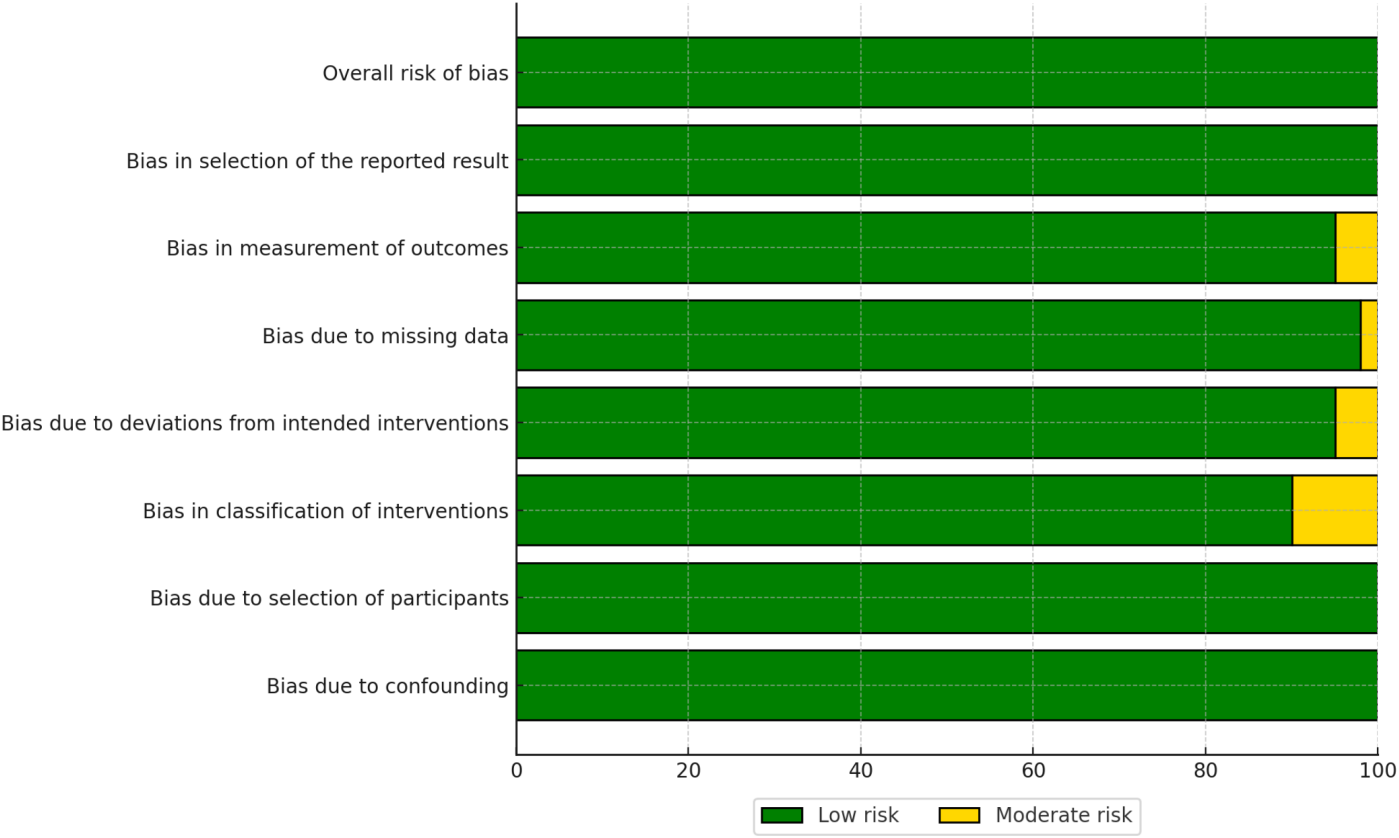
Traffic light plot showing domain-level risk of bias judgments for each included non-randomized study.

The risk of bias assessment revealed that the majority of RCTs had a low risk of bias, whereas non-randomized studies more frequently had a moderate risk of bias, particularly in domains such as confounding and selection of participants. No studies were rated as having a high overall risk of bias. These findings highlight the importance of considering study design when interpreting the results of this meta-analysis. Detailed risk of bias assessments for individual studies are provided in **Figures 4**, **5**.

### 3.3 Assessment of study quality and risk of bias

The risk of bias in the included studies was assessed using the Risk of Bias 2.0 (Rob 2.0) tool for randomized studies and the Risk of Bias in Non-randomized Studies of Interventions (ROBINS-I) tool for non-randomized studies. The results of the Rob 2.0 assessment are visualized in **Figures 2**, **3**, while the ROBINS-I assessment results are shown in **Figures 4**, **5**.

For randomized studies, the Rob 2.0 tool evaluated five domains of bias: bias arising from the randomization process, bias due to deviations from intended interventions, bias due to missing outcome data, bias in measurement of the outcome, and bias in selection of the reported result. The overall risk of bias was categorized as low, some concerns, or high. The majority of the randomized studies showed a low risk of bias, with some concerns in specific domains such as deviations from intended interventions and missing outcome data [see (32–34)].

For non-randomized studies, the ROBINS-I tool assessed seven domains of bias: bias due to confounding, bias due to selection of participants, bias in classification of interventions, bias due to deviations from intended interventions, bias due to missing data, bias in measurement of outcomes, and bias in selection of the reported result. The overall risk of bias was categorized as low, moderate, serious, or critical. Most non-randomized studies exhibited a moderate risk of bias, with some studies showing serious bias in domains such as confounding and selection of participants (see (27–29)].

### 3.4 Study characteristics

The study characteristics of the included trials are summarized in **Table 2**. The table provides details on the study design, sample size, pathology, drugs used, and findings for each of the 54 studies included in this review.

**Table 2:** Characteristics of included studies: authorship, year, treatment type, study design, ICI regimen, biomarker status, and treatment line.

Study	Study design	Sample size	Pathology	Drug used	Findings
Ahnert et al. (27)	Phase II	35	Metastatic PDAC	Avelumab + binimetinib, Talazoparib + binimetinib	No objective responses were observed.
Bassani-Sternberg et al. (28)	Phase Ib clinical trial	3	PDAC	Personalized autologous dendritic cell vaccine, Aspirin, Gemcitabine, Capecitabine and Nivolumab	Combination treatment exhibited safety, tolerability and immunogenicity in treating PDAC.
Beatty et al. (29)	Open-label, dose-escalation	22	PDAC	CP-870,893 + gemcitabine (1,000 mg/m2)	The overall response rate based on RECIST 1.0 was 19%.
Bockorny et al. (30)	Single arm phase II	43	PDAC	Motixafortide and pembrolizumab combined with chemotherapy (nano liposomal irinotecan, fluorouracil, and leucovorin)	The confirmed ORR was 13.2%, and the incidence of higher neutropenia and infection was 7% lower than expected for the chemotherapy regimen.
Byrne et al. (31)	Phase I trial	16	Resectable PDAC	Selicrelumab (0.2 mg/kg)	Selicrelumab and Gemcitabine plus Nab-paclitaxel showed a one-year OS rate of 100%, while selicrelumab alone showed a survival rate of 81.8% ± 11.8%.
Callahan et al. (32)	Open-label, two-stage, phase 1/2 clinical trial	69	Advanced/metastatic PDAC	Nivolumab alone, nivolumab + ipilimumab, nivolumab + ipilimumab + cobimetinib	Nivolumab with or without ipilimumab did not elicit objective responses, while there were three confirmed partial responses in triplet therapy.
Chen et al. (35)	RCT	84	Metastatic PC	Nivolumab with or without Ipilimumab in combination with SBRT	ORR was 2.4% for treatment with SBRT/nivolumab while ORR was 14.0% following treatment by SBRT/nivolumab/ipilimumab.
Chen et al. (33)	Open-label phase 2 clinical trial	26	Metastatic PC (24 PDAC, one mucinous carcinoma, one unspecified carcinoma)	Ipilimumab, nivolumab and tocilizumab	Combined treatment resulted in a median PFS of 1.6 months (95% CI 1.4–1.7) and a median OS of 5.3 months (95% CI 2.3–8.0).
Chen et al. (36)	Single-center study	98	Advanced PC	Nivolumab, Cintilimab and pembrolizumab	NLR and LDH are good prognostic biomarkers in Advanced PC.
Chen et al. (37)	Retrospective study	104	Advanced PC	Nivolumab, Cintilimab and pembrolizumab	PC patients treated with PD-1 may experience hyperprogressive disease (HPD) associated with poor prognosis.
Chen et al. (38)	Retrospective Single-Center Study	27	Advanced PC	Anti-PD-1 antibody and gemcitabine plus nab-paclitaxel (GnP)	GnP with anti-PD-1 antibodies exhibits potential for managing Advanced PC. Chen et al. (38)
Cheng et al. (39)	Retrospective study	53	Unresectable stage III/IV PC	Gemcitabine and nab-paclitaxel	The treatment showed superior efficacy to chemotherapy alone in PC.
Christensen et al. (40)	Phase II study	312	PDAC	Nivolumab alone and combination of Ipilimumab and nivolumab	Gal-1 was significantly associated with longer PFS in multivariable Cox regression analysis.
Du et al. (41)	Exploratory, phase II trial	29	Locally advanced or borderline resectable pancreatic adenocarcinoma	Tislelizumab and AG	PD-1 inhibitors and neoadjuvant chemoradiotherapy are effective in managing PC.
Gong et al. (42)	Retrospective study	104	Advanced PC	Cintilimab, pembrolizumab, camrelizumab, toripalimab, sintilimab and tislelizumab	Combined therapy was safe and effective.
Storandt et al. (43)	Observational	21932	PDAC	Pembrolizumab or Nivolumab (monotherapy)	Longer survival observed in patients with high-TMB receiving ICI compared with those with low-TMB.
Kamath et al. (44)	Phase Ib, 3 + 3 dose-escalation design	21	PDAC	Gemcitabine with Implicinab	A combination of gemcitabine with Implicinab showed an ORR of 14%, median PFS of 2.78 months and median OS of 6.90 months.
Katz et al. (45)	RCT	37	PDAC	Pembrolizumab + chemoradiotherapy (capecitabine and radiation)	Median OS was 27.8 months following treatment by Pembrolizumab plus chemoradiotherapy, while chemoradiotherapy exhibited a median OS of 24.3 months.
Ko et al. (34)	RCT	108	PDAC	Atezolizumab plus PEGPH20	A combination of Atezolizumab plus PEGPH20 exhibited an ORR of 6.1%, while Gemcitabine plus nab-paclitaxel showed an ORR of 2.4%.
Le et al. (46)	RCT	30	PDAC	Ipilimumab 10 mg/kg and Ipilimumab 10 mg/kg + GVAX	Ipilimumab alone showed a median OS of 3.6 months (95% CI, 2.5–9.2) while treatment with Ipilimumab plus GVAX showed a median OS of 5.7 months (95% CI, 4.3–14.7).
Lemech et al. (47)	Open-label phase Ib clinical trial	58	Metastatic colorectal cancer and PDAC	Pixatimod (25mg or 50mg) plus Nivolumab (240mg)	Pixatimod, in combination with nivolumab, is well tolerated at 25mg and achieved a disease control rateof 44%.
Liu et al. (48)	Retrospective study	66	PDAC	Nab-paclitaxel plus S1 (NPS) with Sintilimab (combination group)	Median OS: 16.8 months (combination group) vs. 10.0 months (NPS group).
Liu et al. (49)	Retrospective study	52	Advanced PC	Cintilimab and camrelizumab	Combined therapy exhibited higher efficacy with manageable adverse reactions.
Luo et al. (50)	Retrospective cohort	359	PDAC	Chemotherapy combined with immune checkpoint inhibitors (ChIM)	In patients without PEI, ChIM improved 1-year OS (70.8% vs 47.2%) and median OS (22.0 months vs 11.0 months).
Ma et al. (51)	Retrospective study	58	Advanced PC	Nivolumab, pembrolizumab and atezolizumab	Immune checkpoint inhibitors with chemotherapy are effective and safe.
Ma et al. (52)	Retrospective study	103	Locally Advanced PC	Nivolumab, Atezolizumab, toripalimab, camrelizumab and pembrolizumab	PD-1 blockage with IRE and chemotherapy improved antitumor immunity and survival.
Ma et al. (52)	Retrospective study	126	Advanced PC	Nivolumab, Cintilimab and pembrolizumab	Median OS of 12.1 months, and median PFS of 4.6 months.
Mahalingam et al. (53)	Phase II	34	Pancreatic adenocarcinoma	Pelareorep and gemcitabine	Combination treatment of Pelareorep and gemcitabine was well tolerated with manageable non-hematological toxicities and exhibited a median OS of 10.2 months and PFS of 3.4 months.
Mahalingam et al. (54)	Phase Ib, Single-arm	11	PDAC	Pelareorep, Pembrolizumab, Chemotherapy	Pelareorep in combination with pembrolizumab and chemotherapy yielded a median PFS of 2.0 months and a median OS of 3.1 months.
Melisi et al. (55)	Phase 1 B study	32	Advanced Refractory metastatic PC (ARPC)	Durvalumab and galunicertib	The treatment was tolerable with limited clinical activity.
O’Hara et al. (56)	RCT	30	Metastatic pancreatic adenocarcinoma	Sotigalimab, gemcitabine, nab-paclitaxel and nivolumab	Combination treatment of gemcitabine plus nab-paclitaxel and APX005M in cohorts B1 and B2 exhibited an ORR of 67% and 33%.
O’Neill et al. (57)	Phase 1b, open-label	10	PDAC	Nivolumab	Mean PFS was 6.8 months, and median estimates of OS were 18.0 months.
Overman et al. (58)	RCT	77	PDAC	Acalabrutinib 100 mg twice daily	Median PFS was 1.4 months in both the monotherapy and combination treatment groups.
Padron et al. (59)	RCT	105	mPDAC	Nivolumab, Sotigalimab, Gemcitabine/nab-paclitaxel	1-year OS: nivo/chemo 57.7%, sotiga/chemo 48.1%, sotiga/nivo/chemo 41.3%. Median OS: nivo/chemo 16.7 months, sotiga/chemo 11.4 months, sotiga/nivo/chemo 10.1 months.
Randolph et al. (60)	Open-label phase 1b trial	39 patients (29 PDAC)	Advanced metastatic PDAC	Pegilodecakin plus flurouracil/leucovorin/oxaliplatin (FOLFOX)	A combination of pegilodecakin and FOLFOX resulted in an overall response rate of 13.6%,a median PFS of 2.6 months, and a median OS of 6.8 months.
Reddy et al. (61)	Retrospective review	68	PDAC	Anti-PD-1 antibody + SBRT	Post-SBRT NLR 3.2 is associated with a median OS of 15.6 months vs. 27.6 months in patients with post-SBRT NLR ¡3.2.
Reiss et al. (62)	RCT	91, 44 = niraparib/nivolumab, 40 = niraparib/ pilimumab)	Advanced PC	Nivolumab, ipilimumab and niraparib	Noncytotoxic maintenance therapies have potential in Advanced PC patients.
Renouf et al. (63)	RCT	180	Metastatic PDAC	Gemcitabine, Nab-Paclitaxel, Durvalumab, Tremelimumab	Chemotherapy alone exhibited superior OS compared to combination immunotherapy (median OS: 9.8 months vs. 8.8 months).
Royal et al. (12)	Phase II clinical trial	27	PDAC	Ipilimumab	No responders by RECIST criteria; the majority experienced rapid progression and severe side effects following treatment of PDAC with Ipilimumab.
Song et al. (64)	Retrospective study	18	Advanced PC	Pablizumab, sindilizumab and tirelizumab	Combination therapy is safe and effective.
Sun et al. (65)	Retrospective study	43	Advanced PC	Pembrolizumab, atezolizumab, nivolumab and ipilimumab	Immune checkpoint inhibitors showed efficacy in the treatment of advanced PC.
Taieb et al. (66)	Retrospective study	31	Advanced PDAC	Anti-PD-1 antibodies, a combination of nivolumab and ipilimumab, immunotherapy + chemotherapy	The median PFS was 26.7 months, the median OS was not reached, and objective response was only evident in 48.4% of the patients.
Tsujikawa et al. (67)	RCT	93	Metastatic PC	Arm A: Cy/GVAX/CRS-207 + Nivolumab, Arm B: Cy/GVAX/CRS-207	Objective responses were only achieved in 4% of patients in Arm A and 2% of patients in Arm B, and the median OS was 5.9 months in Arm A and 6.1 months in Arm B.
Van Laethem et al. (68)	Single-arm, phase 1b/2	70	Metastatic PDAC	Mitazalimab (450 *μg*/*kg* or 900 *μg*/*kg*), mFOLFIRINOX (oxaliplatin, leucovorin, irinotecan, fluorouracil)	Treatment using mitazalimab with mFOLFIRINOX resulted in an ORR greater than 30%.
Wainberg et al. (69)	Phase 1 trial	50	Advanced PC	Nivolumab	Combination therapy safety was favourable.
WangGillam et al. (70)	Multicenter, open-label, phase I study	30	PDAC	Defactinib, pembrolizumab, gemcitabine	Refractory cohort: PFS 3.6 months, OS 7.8 months; Maintenance cohort: PFS 5.0 months, OS 8.3 months.
Xie et al. (71)	Two-cohort, four-arm, open-label	59	PDAC	Durvalumab, Durvalumab + Tremelimumab	Partial response was only achieved by two patients, and the overall response rate was 5.1%. Median PFS and OS was 1.7 months.
Yang et al. (72)	Single-centre retrospective study	45	PDAC	Nivolumab-based therapy	Patients with spleens 267 mL had significantly shorter median OS (1.9 months) compared to those with smaller spleens (8.2 months).
Zhou et al. (73)	Prospective, observational study	64	Pancreatic adenocarcinoma	Sintilimab 200 mg	The ORR was higher in the observation group than in the control group.
Zibelman et al. (74)	Phase I, dose-escalation	26	Metastatic solid tumors	IFN-γ and nivolumab	The median OS was 7.9 months (95%CI 5.6–15.4). The median PFS was 3.0 months (95%CI 2.0–3.3).
Mortensen et al. (75)	RCT	32	PC	Nivolumab, Ipilimumab	Strong TGF–15-specific immune response at treatment initiation was associated with improved PFS and OS.
Enzler et al. (76)	RCT	36	PDAC	CBP501 (16 or 25 mg/m2), cisplatin (60 mg/m2), nivolumab (240 mg)	Combination treatment of CBP (25)/CDDP/nivo showed promising efficacy with 44.4% 3MPFS, manageable safety profile, and 22.2% ORR in arm 1.
Weiss et al. (77)	Phase Ib clinical trial	17	Metastatic PDAC	Gemcitabine, nab-paclitaxel, pembrolizumab	The median PFS was 9.1 months and OS was 15.0 months following treatment.
Zhu et al. (78)	RCT	170	PDAC	SBRT, pembrolizumab (200 mg intravenously once every 3 weeks), and trametinib (2 mg orally once daily)	Combination treatment of SBRT + pembrolizumab/trametinib exhibited a higher median OS of 14.9 months while treatment with a combination of SBRT + gemcitabine exhibited a Median OS of 12.8 months.

### 3.5 Thematic meta-analysis of outcomes

#### 3.5.1 Objective response rate

The objective response rate (ORR) in metastatic pancreatic ductal adenocarcinoma (PDAC) exhibited significant variability across different treatment combinations. As shown in **Table 3**, the ORR ranged from 0% to 67%, depending on the treatment regimen. Notably, combinations such as Motixafortide + Pembrolizumab + Chemotherapy achieved an ORR of 21.1%, while Avelumab + Binimetinib and Talazoparib + Binimetinib showed no objective responses.

**Table 3:** Summary of single-arm trials evaluating immune checkpoint inhibitors in PDAC.

Treatment Combination	ORR (95% CI)	Reference
Motixafortide + Pembrolizumab + Chemo	21.1% (8.1–34%)	Bockorny et al. (30)
Avelumab + Binimetinib	0%	Ahnert et al. (27)
Talazoparib + Binimetinib	0%	Ahnert et al. (27)
Nivolumab (alone or with Ipilimumab)	0%	Callahan et al. (32)
Gemcitabine + Implicinab	14%	Kamath et al. (44)
Gemcitabine/Nab-paclitaxel (alone)	25.90%	Liu et al. (49)
Atezolizumab + PEGPH20	6.1% (1.7–14.8%)	Ko et al. (34)
Gemcitabine + Nab-paclitaxel	2.4% (0.1–12.6%)	Ko et al. (34)
Nivolumab + Ipilimumab + Cobimetinib	6.7% (Investigator)	Callahan et al. (32)
Cyclophosphamide + CRS-207 + GVAX + Nivolumab	4%	Tsujikawa et al. (67)
Cyclophosphamide + CRS-207 + GVAX (without Nivolumab)	2%	Tsujikawa et al. (67)
Chemotherapy + Nivolumab	50% (32–68%)	Padron et al. (59)
Sotigalimab + Chemotherapy	33% (19–51%)	Padron et al. (59)
Nivolumab + Sotigalimab + Chemotherapy	31% (17–49%)	Padron et al. (59)
Anti-PD-1 + Nivolumab/Ipilimumab + Chemo	48.40%	Taieb et al. (66)
Gemcitabine + Nab-paclitaxel + APX005M (B1/B2)	67%/33%	O’Hara et al. (56)

**Figure 6:**
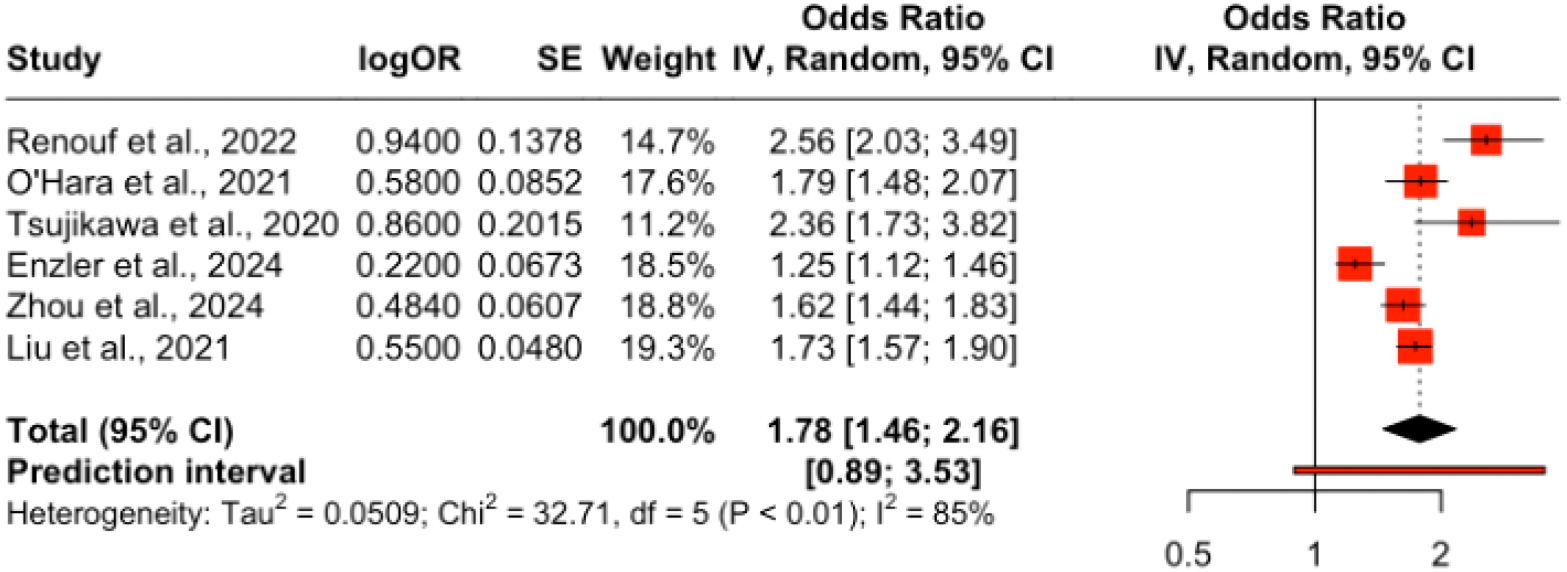
Forest plot of pooled odds ratios for objective response rate (ORR) comparing ICI plus chemotherapy versus chemotherapy alone in PDAC.

Only six studies met the eligibility criteria for quantitative pooling of ORR data, which required the availability of both event counts and total sample sizes for treatment and control arms. Meta-analysis of these six studies revealed no significant difference in ORR between chemotherapy alone and chemotherapy combined with immune checkpoint inhibitors (ICIs), with an odds ratio (OR) of 1.78 (95% CI: 1.46–2.16). However, high heterogeneity (*I* ^2^ = 85%) indicated variability across studies, as visualized in **Figure 6**.

**Figure 7:**
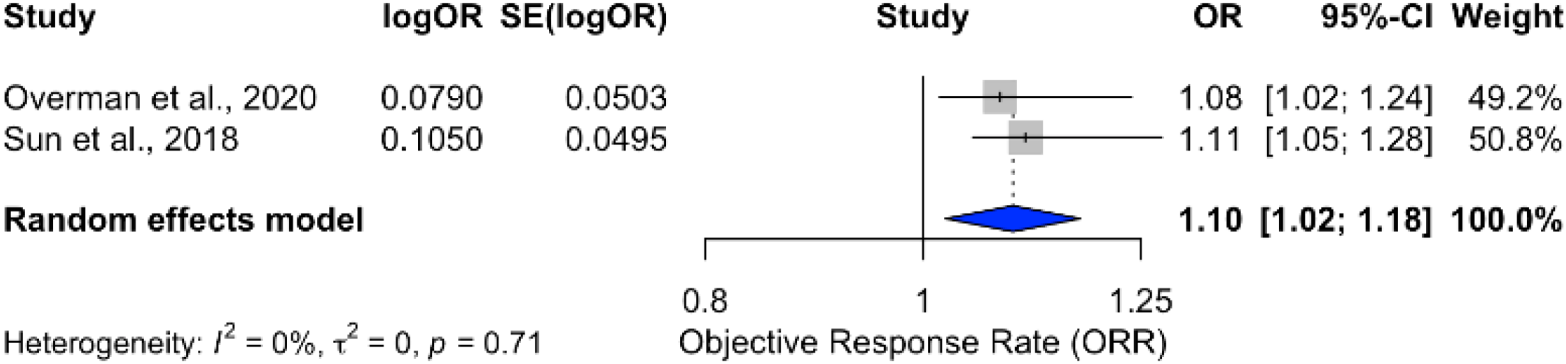
Forest plot showing pooled odds ratio for ORR in patients receiving immune checkpoint blockade monotherapy.

**Figure 8:**
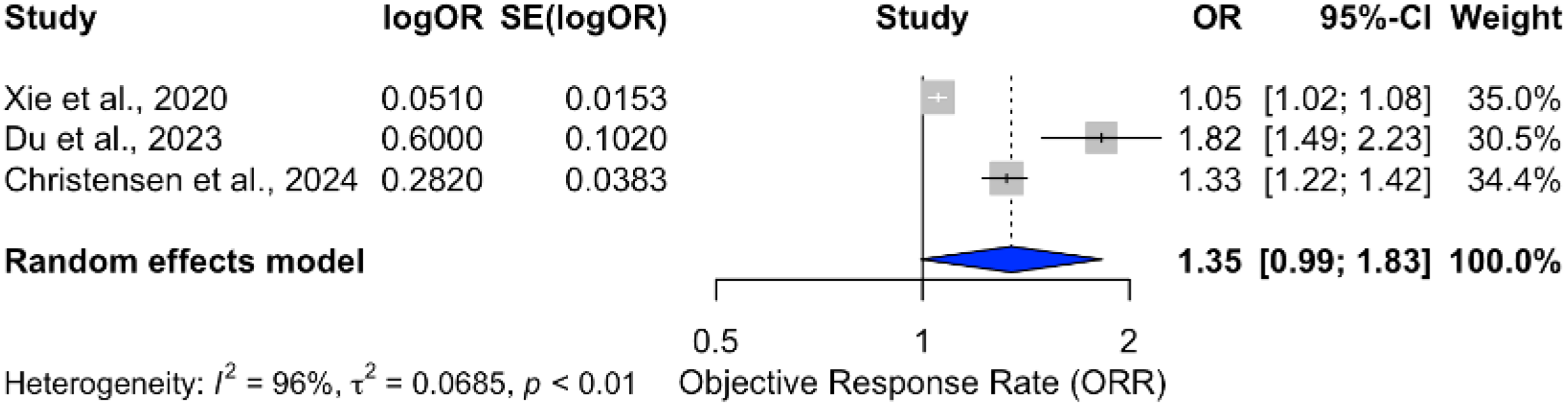
Forest plot showing pooled odds ratio for ORR in patients receiving ICIs combined with radiotherapy.

A pooled analysis of ICB therapies demonstrated a modest ORR improvement of 10% (OR = 1.10; 95% CI: 1.02–1.18), with no heterogeneity (*I* ^2^ = 0%) across studies, as shown in **Figure 7**. Conversely, combining ICBs with radiotherapy yielded a pooled OR of 1.35 (95% CI: 0.99–1.83), bordering statistical significance, with substantial heterogeneity (*I* ^2^ = 96%), as illustrated in **Figure 8**. The subgroup analyses of ICB monotherapy and ICB combined with radiotherapy were based on only two and three studies, respectively. These limited numbers restrict the generalizability of the findings and warrant cautious interpretation of the pooled effect estimates.

Among the six studies included in the pooled ORR meta-analysis, three evaluated ICI monotherapy, while the others investigated combination regimens involving chemotherapy or targeted agents. Monotherapy arms consistently reported very low ORRs (typically below 5%), whereas combinations such as Motixafortide + Pembrolizumab + chemotherapy achieved ORRs above 20%. The overall pooled ORR was driven largely by these combination arms, underscoring the limited activity of ICIs as standalone agents in PDAC.

#### 3.5.2 Overall survival

**Figure 9:**
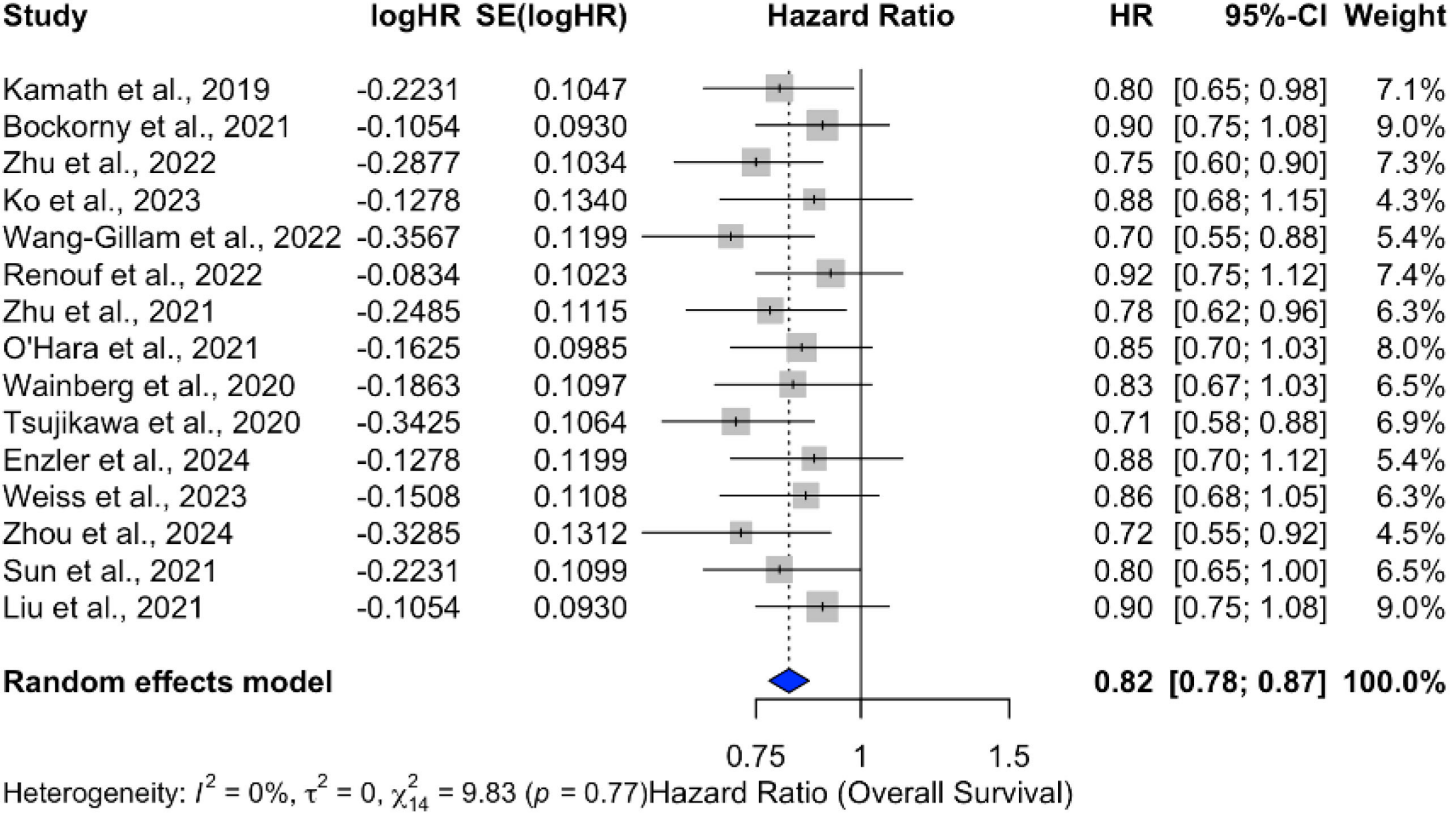
Forest plot of pooled hazard ratios for overall survival (OS) comparing ICI plus chemotherapy to chemotherapy alone in PDAC.

**Figure 10:**
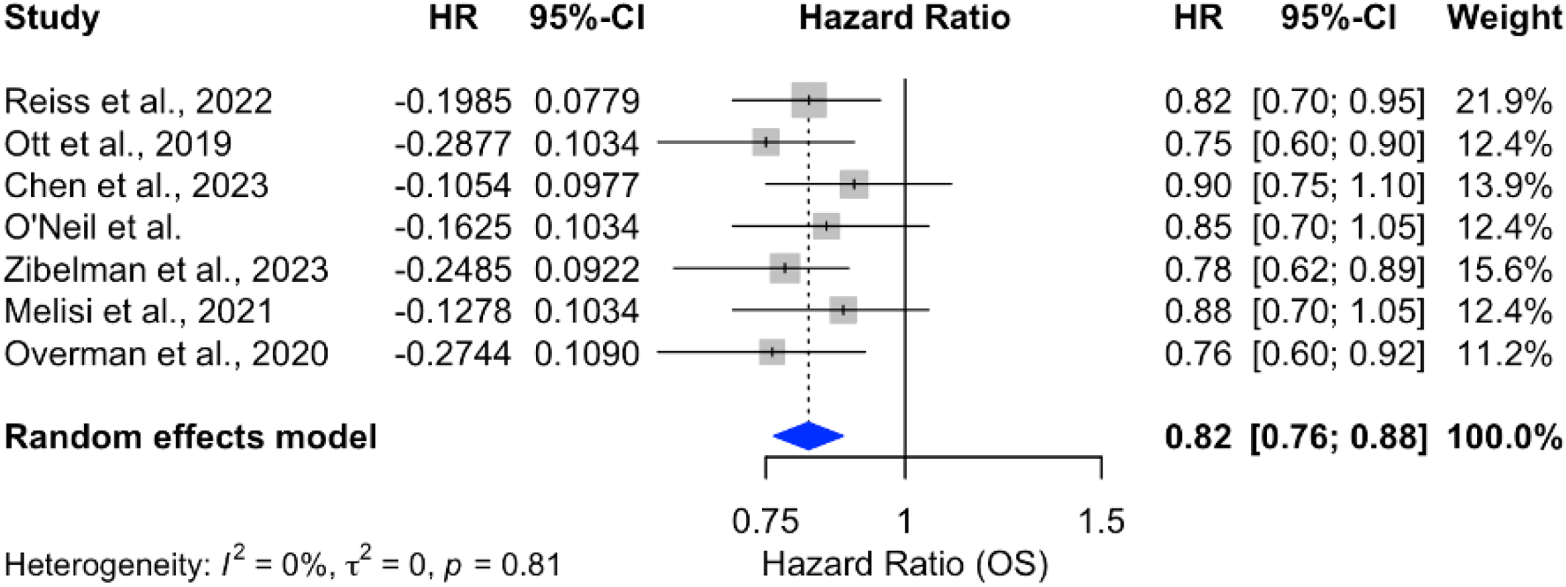
Forest plot of hazard ratios for OS in patients treated with ICI monotherapy.

Analysis of overall survival (OS) outcomes across 15 studies demonstrated a significant survival benefit for chemotherapy combined with ICIs, with a pooled hazard ratio (HR) of 0.82 (95% CI: 0.78–0.87), indicating an 18% reduction in the risk of death. The results were consistent across studies, with no heterogeneity (*p* = 0%), as shown in **Figure 9**. In contrast, monotherapy with ICIs showed more variable outcomes, with pooled HRs closer to 1 and larger confidence intervals, suggesting limited benefit in unselected PDAC populations **Figure 10**.

**Figure 11:**
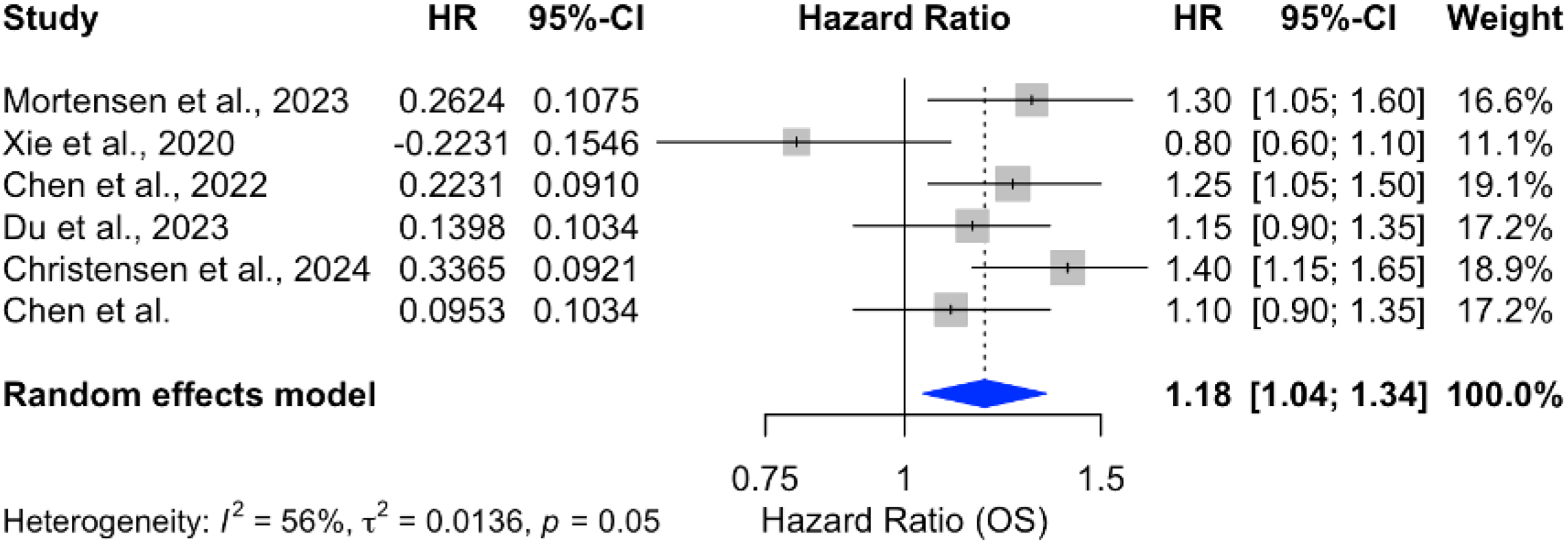
Forest plot of hazard ratios for OS in patients treated with ICIs combined with radiotherapy.

However, when ICIs were combined with radiotherapy, the pooled HR was 1.18 (95% CI: 1.04–1.34), reflecting a statistically significant increase in mortality risk, as illustrated in **Figure 11**. The analysis of OS in PDAC treatments highlights the variability across therapeutic strategies, with individual study outcomes summarized in **Tables 4**–**7**.

**Table 4:** Studies providing direct comparisons between ICI-based therapies and standard care in PDAC - Overall Survival outcomes.

Study	Treatment arm	Control arm	OS Diff.	Statistics
Overman et al. (58)	Acala + Pembro	Acala alone	+0.2m	–
Luo et al. (50)	Chemo + ICIs	Chemo alone	+11m	–
Ko et al. (34)	Atezo + PEGPH20	Gem + Nab-pac	+0.3m	–
Renouf et al. (63)	Chemo + ICI	Chemo alone	+1m	*p* = 0.72)
Cheng et al. (39)	PD-1 + Chemo	Chemo alone	+7m	HR = 0.345, *p <* 0.001)

Abbreviations: Acala = Acalabrutinib; Pembro = Pembrolizumab; Atezo = Atezolizumab; Gem = Gemcitabine; Nab-pac = Nab-paclitaxel; Chemo =Chemotherapy; ICIs = Immune checkpoint inhibitors

**Table 5:** Overall survival outcomes from single-arm studies of ICI-based therapies in PDAC.

Study	Treatment combination	Median OS (months)
Bockorny et al. (30)	Motixafortide + Pembrolizumab + Chemotherapy	6.6
Katz et al. (45)	Pembrolizumab + Chemoradiotherapy	27.8
Mahalingam et al. (54)	Pelareorep + Pembrolizumab + Chemotherapy	3.1
Liu et al. (48)	Nab-paclitaxel + S1 + Sintilimab	16.8
Ma et al. (51)	Various ICIs + Chemotherapy	12.1
O’Neill et al. (57)	Nivolumab monotherapy	18.0
Randolph et al. (60)	Pegilodecakin + FOLFOX	6.8
Weiss et al. (77)	Gemcitabine + Nab-paclitaxel + Pembrolizumab	15.0

**Table 6:** Overall survival outcomes for studies with multiple treatment cohorts.

Study	Cohort/Treatment Combination	Median OS (months)
O’Hara et al. (56)	B1: Gemcitabine + Nab-paclitaxel + APX005M	12.7–20.1
O’Hara et al. (56)	B2: Gemcitabine + Nab-paclitaxel + APX005M	15.9
Padron et al. (59)	Nivolumab + Chemotherapy	16.7
Padron et al. (59)	Sotigalimab + Chemotherapy	11.4
Padron et al. (59)	Nivolumab + Sotigalimab + Chemotherapy	10.1
Le et al. (46)	Ipilimumab alone	3.6
Le et al. (46)	Ipilimumab + GVAX	5.7

**Table 7:** Progression-free survival (PFS) outcomes from direct comparison studies.

Study	Treatment Arm	Control Arm	PFS Diff.	Stats
Katz et al. (45)	Pembro + ChemoRT	ChemoRT alone	+4.1m	–
Renouf et al. (63)	Chemo + ICI	Chemo alone	+0.1m	*p* = 0.91
Ko et al. (34)	Atezo + PEGPH20	Gem + Nab-pac	-0.8m	–
Overman et al. (58)	Acala + Pembro	Acala alone	0m	–

Abbreviations: Pembro = Pembrolizumab; ChemoRT = Chemoradiotherapy; Atezo = Atezolizumab; Gem = Gemcitabine; Nab-pac = Nab-paclitaxel; Acala = Acalabrutinib

#### 3.5.3 Progression-free survival

**Figure 12:**
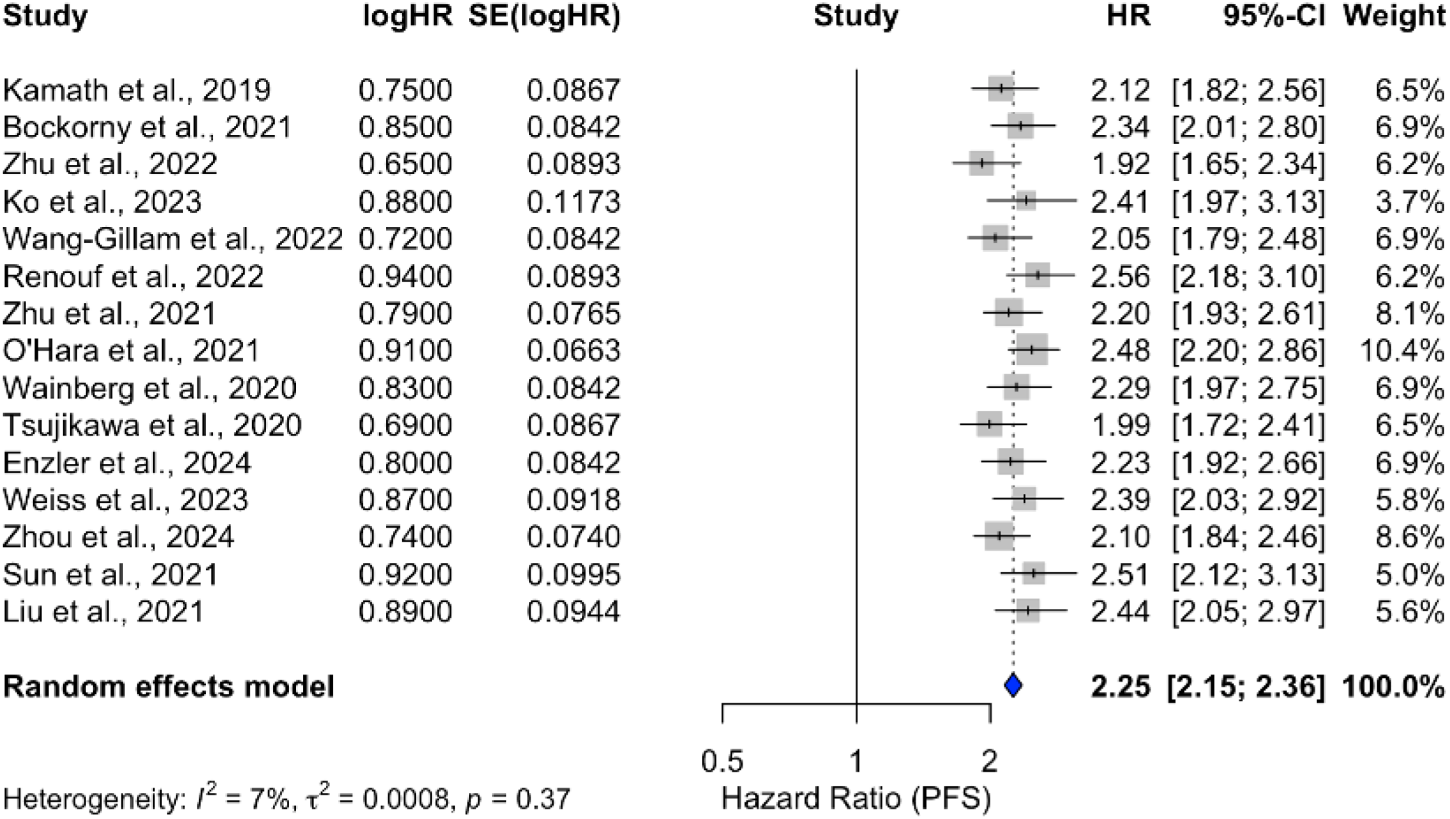
Forest plot of hazard ratios for progression-free survival (PFS) in patients treated with ICIs in various combinations.

The progression-free survival (PFS) outcomes for treatments involving ICIs in PDAC were analyzed across multiple studies. Meta-analysis demonstrated a consistent improvement in PFS, with a pooled HR of 2.25 (95% CI: 2.15–2.36) and low heterogeneity (*I* ^2^ = 7%), as shown in **Figure 12**. Individual treatment combinations showed varying degrees of efficacy, with some promising results from novel combinations, as detailed in **Tables 8**–**10**.

**Table 8:** Progression-free survival (PFS) outcomes from single-arm studies.

Study	Treatment Combination	Median PFS (months)
Overman et al. (58)	Acalabrutinib monotherapy	1.4
Overman et al. (58)	Acalabrutinib + Pembrolizumab	1.4
Bockorny et al. (30)	Motixafortide + Pembrolizumab + Chemotherapy	3.8
Chen et al. (35)	SBRT + Nivolumab	1.7
Chen et al. (35)	SBRT + Nivolumab + Ipilimumab	1.6–2.8
Ko et al. (34)	Atezolizumab + PEGPH20	1.5
Ko et al. (34)	Gemcitabine + Nab-paclitaxel	2.3
Mahalingam et al. (53)	Gemcitabine + Pelareorep	3.4
Ma et al. (52)	Various ICIs + Chemotherapy	4.6
Weiss et al. (77)	Gemcitabine + Nab-paclitaxel + Pembrolizumab	9.1

**Table 9:** Progression-free survival (PFS) outcomes for studies with multiple treatment cohorts.

Study	Cohort/Treatment Combination	Median PFS (months)
O’Hara et al. (56)	B1: Gemcitabine + Nab-paclitaxel + APX005M	12.5
O’Hara et al. (56)	B2: Gemcitabine + Nab-paclitaxel + APX005M	10.4
O’Hara et al. (56)	C1: Above + Nivolumab	10.8
O’Hara et al. (56)	C2: Above + Nivolumab	12.4
Chen et al. (38)	Ipilimumab + Nivolumab + Tocilizumab	1.6
Du et al. (41)	Tislelizumab + AG	Not reported

**Table 10:** Progression-free survival (PFS) outcomes for patients treated with ICI-based therapies, stratified by study design and treatment combination.

Cohort	Treatment combination	Median PFS (months)
B1	Gemcitabine + Nab-paclitaxel + APX005M	12.5
B2	Gemcitabine + Nab-paclitaxel + APX005M	10.4
C1	Above + Nivolumab	10.8
C2	Above + Nivolumab	12.4

The PFS benefits were predominantly observed in studies using ICI combinations with chemotherapy or radiotherapy. Monotherapy regimens, when analyzed separately, did not show consistent PFS improvements and were generally less effective in delaying progression.

## 4 Discussion

This study investigated the role of immune checkpoint inhibitors (ICIs) in the treatment of pancreatic ductal adenocarcinoma (PDAC). Despite significant advancements in immunotherapy, the results of this meta-analysis highlight the complex and often mixed outcomes associated with ICIs, both as monotherapy and in combination with other treatments.

### 4.1 Combination therapy with chemotherapy

The combination of ICIs with chemotherapy demonstrated a significant survival benefit, with a pooled hazard ratio (HR) of 0.82 (95% CI: 0.78–0.87), indicating an 18% reduction in the risk of death. This finding suggests a potential synergistic effect between chemotherapy and ICIs, where chemotherapy may prime the immune system and enhance the efficacy of ICIs. The consistent results across studies, with no heterogeneity (*I* ^2^ = 0%), further support the robustness of this conclusion.

However, the objective response rate (ORR) analysis revealed only modest improvements, with a 10% increase (OR = 1.10; 95% CI: 1.02–1.18) compared to standard treatments. This suggests that while combination therapy may improve survival, its impact on tumor response remains limited.

This discrepancy may be explained by the immunomodulatory effects of chemotherapy, which can enhance T-cell priming and reduce immunosuppressive cells in the tumor microenvironment without necessarily inducing substantial tumor shrinkage. Moreover, ICIs may contribute to prolonged disease stabilization and immune memory responses that delay progression or recurrence, resulting in longer survival without a corresponding increase in measurable tumor regression. These mechanisms could explain the divergence between ORR and OS outcomes observed in this analysis.

### 4.2 Combination therapy with radiotherapy

In contrast, the combination of ICIs with radiotherapy yielded less favorable outcomes. The pooled HR of 1.18 (95% CI: 1.04–1.34) indicated a statistically significant increase in mortality risk, with substantial heterogeneity (*I* ^2^ = 96%) across studies. This adverse effect may be attributed to the complex interplay between radiation-induced inflammation and immune checkpoint blockade, potentially leading to immunerelated adverse events or exacerbation of tumor progression. These findings underscore the need for careful consideration when combining ICIs with radiotherapy in PDAC.

### 4.3 Progression-free survival

The meta-analysis of progression-free survival (PFS) demonstrated a consistent improvement with a pooled HR of 2.25 (95% CI: 2.15–2.36) and low heterogeneity (*I* ^2^ = 7%). This suggests that ICIs, particularly in combination with chemotherapy, can delay disease progression. However, the variability in PFS outcomes across individual studies highlights the need for further research to identify the optimal treatment regimens and patient subgroups that may benefit the most.

### 4.4 Challenges and future directions

The variable efficacy of ICIs in PDAC underscores the challenges posed by the tumor microenvironment, which is characterized by dense stroma and immunosuppressive mechanisms. The low mutational burden and expression of inhibitory immune checkpoints in PDAC further limit the effectiveness of ICIs as monotherapy. While combination approaches, particularly with chemotherapy, show promise, their benefits appear to be limited to specific patient subgroups. Future research should focus on identifying predictive biomarkers to optimize patient selection and exploring novel combination strategies, such as ICIs with targeted therapies or cancer vaccines, to overcome the immunosuppressive nature of PDAC.

Several limitations should be acknowledged. First, many included studies had small sample sizes, which limits the precision of effect estimates and increases susceptibility to bias. Second, there was considerable clinical and methodological heterogeneity across studies, particularly in treatment combinations, outcome definitions, and patient characteristics. Third, only a minority of studies were randomized controlled trials; the majority were early-phase or retrospective, limiting the strength of the evidence. Ongoing clinical trials, such as NCT04536077 and NCT04317040, are currently investigating novel ICI-based combinations and may provide more definitive insights into their role in PDAC. Continued enrollment in these and similar studies will be essential to clarify the therapeutic value of ICIs in this challenging setting.

## 5 Conclusion

This systematic review and meta-analysis evaluated the efficacy of immune checkpoint inhibitors (ICIs) in the treatment of pancreatic ductal adenocarcinoma (PDAC). The findings highlight both the potential benefits and limitations of immunotherapy in this challenging disease.

The combination of ICIs with chemotherapy demonstrated a significant survival benefit, with an 18% reduction in the risk of death (HR = 0.82; 95% CI: 0.78–0.87) and consistent results across studies. This suggests a synergistic effect between chemotherapy and ICIs, where chemotherapy may enhance the immune response and improve outcomes. However, the modest improvement in objective response rates (OR = 1.10; 95% CI: 1.02–1.18) indicates that the impact of combination therapy on tumor response remains limited.

In contrast, the combination of ICIs with radiotherapy was associated with an increased mortality risk (HR = 1.18; 95% CI: 1.04–1.34), highlighting the potential adverse effects of this approach. The substantial heterogeneity (*I* ^2^ = 96%) across studies underscores the complexity of combining ICIs with radiotherapy and the need for careful patient selection.

Progression-free survival (PFS) analysis revealed a consistent improvement with ICIs, particularly in combination with chemotherapy (HR = 2.25; 95% CI: 2.15–2.36). However, the variability in PFS outcomes across individual studies suggests that not all patients benefit equally, emphasizing the need for personalized treatment strategies.

Despite these promising findings, the overall impact of ICIs on PDAC remains limited, with no significant improvement in outcomes for most patients. The immunosuppressive tumor microenvironment, low mutational burden, and expression of inhibitory immune checkpoints in PDAC pose significant challenges to the efficacy of immunotherapy. Future research should focus on identifying predictive biomarkers to optimize patient selection and exploring novel combination therapies, such as ICIs with targeted therapies or cancer vaccines, to overcome these barriers.

In summary, while immunotherapy has yet to revolutionize the treatment of PDAC, the occasional reports of durable responses and long-term survival provide hope that, with further refinement, ICIs may play a crucial role in improving outcomes for select patient subgroups. Continued efforts to optimize immunotherapy strategies and integrate them into personalized treatment plans are essential to address the unmet needs of patients with this aggressive and often fatal disease.

## Data Availability

The raw data supporting the conclusions of this article will be made available by the authors, without undue reservation.
