# Supplementary material for: Immune checkpoint inhibitors in pancreatic adenocarcinoma: a systematic review and meta analysis of clinical outcomes
Source: Front Oncol. 2025 Aug 8;15:1569884. doi: 10.3389/fonc.2025.1569884 (PMC12370491; doi:10.3389/fonc.2025.1569884)
Supplement: Supplementary file 1 [file DataSheet1.pdf]

Database	Search Strategy
PubMed	(”pancreatic cancer”[MeSH Terms] OR ”pancreatic neoplasms”[MeSH Terms] OR ”pancreatic ductal adenocarcinoma” OR ”PDAC” OR ”pancreatic tumor”) AND (”immune checkpoint inhibitors” OR ”checkpoint blockade” OR ”PD-1” OR ”PD-L1” OR ”CTLA-4” OR ”nivolumab” OR ”pembrolizumab” OR ”atezolizumab”)
Embase	(’pancreatic cancer’/exp OR ’pancreatic ductal adenocarcinoma’:ab,ti OR PDAC:ab,ti) AND (’immune checkpoint inhibitor’/exp OR ’checkpoint blockade’:ab,ti OR ’PD-1’:ab,ti OR ’PD-L1’:ab,ti OR ’CTLA-4’:ab,ti)
Scopus	TITLE-ABS-KEY(”pancreatic ductal adenocarcinoma” OR ”pancreatic cancer” OR PDAC) AND TITLE-ABS-KEY(”immune checkpoint inhibitors” OR PD-1 OR PD-L1 OR CTLA-4 OR nivolumab OR pembrolizumab)
Web of Science	TS=(”pancreatic ductal adenocarcinoma” OR ”pancreatic cancer” OR PDAC) AND TS=(”immune checkpoint inhibitors” OR ”PD-1” OR ”PD-L1” OR ”CTLA-4” OR ”nivolumab” OR ”pembrolizumab”)
ClinicalTrials.gov	Condition or disease: ”Pancreatic Cancer” AND Other terms: ”immune checkpoint inhibitor” OR ”PD-1” OR ”PD-L1” OR ”CTLA-4”

Table 2: Supplemental Table 1: Full search strategies used for all databases. Searches were conducted through March 15, 2024.
